# LONG-TERM MOBILITY DEVICE USE AND SOCIAL WELL-BEING AMONG COMMUNITY-DWELLING OLDER ADULTS IN THE UNITED STATES

**DOI:** 10.1093/geroni/igae098.2811

**Published:** 2024-12-31

**Authors:** Xinran Liu, Sara Baumann, Andrea Rosso, Elizabeth Venditti, Yao Yao, Steven Albert

**Affiliations:** Peking University, China (People’s Republic); University of Pittsburgh, Pittsburgh, Pennsylvania, United States; University of Pittsburgh, Pittsburgh, Pennsylvania, United States; University of Pittsburgh, Pittsburgh, Pennsylvania, United States; Peking University, Beijing, Beijing, China (People’s Republic); University of Pittsburgh, Pittsburgh, Pennsylvania, United States

## Abstract

The prevalent use of mobility devices among older adults is largely driven by the increased incidence of mobility disability in later life. While these devices are designed to enhance activities and participation for individuals with mobility disability, their long-term effects on social well-being remain unclear. This study assesses whether mobility device use contributes to improved activities, participation, and life satisfaction among community-dwelling older adults over time, comparing users with their non-user counterparts. We utilized data from the National Health and Aging Trends Study (NHATS) waves 1-9 (2010-2019) to identify incident mobility device users with over two years of experience. These users were matched on a 1:1 basis with non-users by age (±5 years), gender, race, and baseline Short Physical Performance Battery scores (±1 point). Follow-up assessments were conducted at the time of incident use, and 1- and 2-year later. Guided by the NHATS late-life disability framework, we applied mixed-effects models, adjusting for personal and environmental factors, health status, body impairments, functional capacity, and accommodations. The results indicate that mobility device users have overall significantly lower participation in paid work compared to non-users (p< 0.05). Over time, mobility device use was associated with decreased driving but increased reliance on rides from others, and more frequent visits with family and friends (ps< 0.05). Notably, older mobility device users exhibited lower life satisfaction in the long term (p< 0.05). These findings underscore the critical need to address the social well-being of older adults who use mobility devices in communities to improve their overall quality of life.

